# Waiting time for scheduled outpatient specialist consultations by access pathway in public hospitals in Ecuador

**DOI:** 10.1371/journal.pone.0349550

**Published:** 2026-09-17

**Authors:** Marcelo Armijos Briones, Emilia Diaz Cercado, Oscar Marcillo-Toala, Patricia Estefanía Ayala Aguirre, Pablo Lenin Benitez Sellan, Antonio Lanata-Flores, Nicole Armijos Bazurto

**Affiliations:** 1 School of Dentistry, Universidad Espíritu Santo, Samborondón, Ecuador; 2 Pvalor SAS, Portoviejo, Ecuador; Shahid Beheshti University of Medical Sciences, IRAN, ISLAMIC REPUBLIC OF

## Abstract

**Objective:**

To quantify waiting time in days for scheduled outpatient specialist consultations and to compare waiting time between standardized and non-standardized access pathways in Ecuadorian public hospitals.

**Methods:**

We analyzed hospital-based survey data from Ecuadorian public hospitals, restricted to adults attending a scheduled outpatient specialist consultation (n = 4,436). Emergency care, unscheduled urgent visits, procedures, and follow-up visits were excluded by design. Access pathway was classified from participants’ self-report as standardized (institutional or system-based) or non-standardized (informal or non-system-based). Waiting time, defined as the number of days between obtaining the appointment and attending the consultation, was compared using the Mann–Whitney U test. Sociodemographic correlates of non-standardized access were examined using adjusted logistic regression, and adjusted median differences were estimated using quantile regression (τ = 0.50). Effect modification by specialty referral requirement was assessed in a single median regression model using an interaction between access pathway and referral-requirement stratum.

**Results:**

Non-standardized access was associated with shorter waiting times than standardized access. In median regression adjusted for sociodemographic covariates, specialty, and hospital, with hospital-clustered inference, non-standardized access was associated with a 3.6-day shorter conditional median waiting time (95% CI −6.2 to −1.0). Adjusted stratum-specific contrasts were −7.9 days (95% CI −13.7 to −2.1) in direct-access specialties and −3.1 days (95% CI −5.9 to −0.2) in referral-required specialties; however, the interaction between access pathway and referral-requirement stratum was not statistically significant (p = 0.136).

**Conclusion:**

Among patients who attended a scheduled outpatient specialist consultation in Ecuadorian public hospitals, non-standardized access was associated with shorter waiting times. Although the adjusted point estimate was larger in direct-access specialties, the formal interaction test did not provide clear evidence that the association differed according to specialty referral requirement.

These findings suggest that, within routine outpatient care, parallel access pathways may shape timeliness and warrant greater transparency in appointment allocation and referral coordination.

## Introduction

Timely access to specialist care is a core dimension of effective health system performance and quality. Delays might result in avoidable suffering, disease progression, and inefficient use of resources [1]. Conceptual frameworks emphasized that access to care was not only determined by service availability, but also how the population could identify needs, seek care, reach services, and obtain appropriate care without delay [2]. Waiting time is often used as a non-monetary rationing mechanism in publicly financed systems, however the burden of waiting is not evenly distributed across socioeconomic groups [3–5]. Evidence from different high-income settings showed that income and education have been related with shorter waiting times for specialist or planned care, challenging the assumption that waiting lists automatically delivered “*equal treatment for equal need*” [4,6,7].

In Latin America, health-system reforms have expanded coverage and benefits, yet fragmentation and persistent inequalities continue to shape access to specialist care. Delays in referral pathways, weak coordination between levels of care, and administrative barriers remain important challenges in public healthcare networks. However, patient-level empirical evidence on how standardized and non-standardized access pathways relate to waiting time for outpatient specialist consultations remains limited in the region. Evidence suggests that incomplete referral information, limited feedback from specialists, and inadequate communication tools are the most common shortcomings in public health networks, which may contribute to delays and inefficiencies in the outpatient referral process [8]. Recent regional analyses have highlighted persistent operational barriers affecting timely access to care in Latin America and the Caribbean [9].

A key reason this gap matters is that “access” is not only defined by standard rules. A classic work conceptualized access as the fit between patients and health systems across dimensions including availability, accessibility, and accommodation [2,10]. When standard (system-based) pathways are being constrained by capacity, administrative complexity, or weak coordination, patients might seek non-standardized (non-system-based) routes, through personal networks or other mechanisms, to obtain appointments with shorter intervals. If such non-standardized routes systematically reduced waiting time for some individuals, then timeliness might become partly determined by social connections rather than clinical needs, widening inequities even within a publicly financed system.

In Ecuador, the Comprehensive Health Care Model (Modelo de Atención Integral de Salud, MAIS) organizes care across levels of complexity and formally relies on a referral and counter-referral subsystem [11]. Within this framework, outpatient specialist care in public hospitals offers an informative setting to examine waiting time and access pathways, particularly because some specialties may be accessed in routine practice with weaker referral constraints than others. The aim of the present study was to quantify waiting time in days for scheduled outpatient specialist consultations and to compare waiting time between standardized and non-standardized access pathways. We also examined sociodemographic correlates of non-standardized access and explored whether the association between access pathway and waiting time differed between direct-access and referral-required specialties.

## Methods

### Study design and setting

This was a cross-sectional observational study based on a hospital survey conducted in public hospitals in Ecuador. The study design, site selection, field procedures, and questionnaire development were described in a published protocol [12]. The present manuscript reports a focused analysis of waiting time and access pathways for scheduled outpatient specialist consultations.

### Participants and eligibility

Participants were interviewed outside public hospitals before entering the outpatient specialist area. Eligible participants were adults attending a scheduled outpatient specialist consultation in the public hospital network. Emergency care, unscheduled urgent visits, appointments for procedures, and follow-up visits with the same specialist were excluded by design because the present analysis focused on first scheduled outpatient specialist consultations.

### Data collection

After informed consent, trained interviewers administered the questionnaire verbally and recorded responses in a structured Google Forms survey, which uploaded the information to a centralized database in real time. Interviewers were deployed across the country, except in the Galápagos Islands for logistical reasons. Data collection took place outside hospitals because institutional authorization for in-hospital recruitment was not available; as interviews were conducted in public space before outpatient entry, this procedure did not require additional authorization from the Ministry of Public Health. Data collection outside of public hospitals began on 29/07/2024 and ended on 12/10/2024.

### Exposure definition

Standardized access was defined as obtaining the appointment through the institutional referral pathway of the public health system. According to the original study protocol, this included referral from a doctor at a health center, referral from a specialist at this or another hospital, and appointment scheduling at the hospital or by telephone. Non-standardized access was defined as obtaining the appointment through informal or non-system-based mechanisms, including through a friend or family member or through a person who was not a friend or family member. Throughout the manuscript, non-standardized access refers to these informal or non-standardized mechanisms.

### Outcome definition

Waiting time was defined as the self-reported number of days between the date on which the outpatient specialist appointment was obtained and the date on which that scheduled consultation was attended.

### Covariates

Self-reported covariates included age, sex, education level, geographic region (Sierra, Coast, Amazon), ethnicity, area of residence (urban or rural), and household income. Income was recorded numerically and categorized into sample-based quintiles (Q1 lowest to Q5 highest).

### Specialty stratification

For the stratified analyses, specialties were grouped a priori according to the referral requirement in routine practice. Dentistry, General Medicine, Family Medicine, and Psychology were classified as direct-access specialties, since these specialties are directly accessible in hospitals; that is, a referral is not required to access them. All other specialties were classified as referral-requiring specialties.

### Statistical analysis

Participant characteristics were summarized overall and by access pathway. Continuous variables were described using means and standard deviations when approximately symmetric, and medians with interquartile ranges (IQR) for skewed distributions. Categorical variables were summarized using counts and percentages.

Waiting time distributions were compared between access pathways using the Mann–Whitney U test, consistent with the non-normal distribution of waiting time.

To examine sociodemographic correlates of non-standardized access, we fitted a binary logistic regression model with non-standardized access as the dependent variable and reported crude and adjusted odds ratios (ORs) with 95% confidence intervals (CIs). The adjusted model included age, sex, education, geographic region, ethnicity, household income quintile, and area of residence.

To estimate the adjusted association between access pathway and waiting time in absolute days while accommodating right-skewness, we fitted a median regression model (τ = 0.50) with waiting time as the outcome and access pathway as the primary exposure. The model included age, sex, education, geographic region, ethnicity, household income quintile, area of residence, and fixed effects for individual specialty and hospital. We reported the adjusted conditional median difference (non-standardized minus standardized) with its 95% confidence interval.

To assess whether the association between access pathway and waiting time differed according to specialty referral requirement, we fitted a single median regression model that included an interaction between access pathway and referral-requirement stratum. This interaction model contained the same sociodemographic covariates and hospital and individual-specialty fixed effects as the primary model. Because referral requirement was determined by individual specialty, its main effect was absorbed by the specialty fixed effects. Adjusted contrasts for non-standardized versus standardized access were estimated within each stratum, and effect modification was assessed using the coefficient and p value of the interaction term. Descriptive medians and interquartile ranges by access pathway were also reported within each stratum.

Because participants were clustered within hospitals, all regression models used hospital-clustered sandwich variance estimators. We applied a CR1 finite-sample correction and based confidence intervals and hypothesis tests on a t distribution with 25 degrees of freedom, corresponding to 26 hospital clusters. For the median-regression models, the clustered sandwich covariance matrix was based on quantile scores and a residual-density estimate.

All 4,436 participants had complete data for the variables included in the final regression models and were retained in these analyses.

Because this study focused exclusively on scheduled outpatient specialist consultations, emergency presentations and unscheduled urgent care were excluded by design. In the Ecuadorian public system, patients with acute urgent conditions are generally directed to emergency services rather than managed through routine outpatient scheduling. However, some scheduled outpatient consultations may still differ in perceived clinical priority, and this factor was not directly measured.

### Ethics

This study was approved by the Ethics Committee for Research in Human Subjects of the Portoviejo Higher Technological University Institute (CEISH-ITSUP) under code 1718079732. The committee is registered with the Ministry of Public Health of Ecuador and with the Office for Human Research Protections under number IRB00014260. All participants provided informed consent before the interview. No directly identifiable personal information was collected.

## Results

The analytic sample comprised 4,436 adults who attended a scheduled outpatient specialist consultation in public hospitals in Ecuador. Overall, 1,431 participants (32.3%) reported standardized access and 3,005 (67.7%) reported non-standardized access. The mean age was 43·22 years (SD 15·38), and women accounted for 63·5% of the sample. Most respondents reported secondary education (51·5%), followed by primary education (26·9%), and third-level education (17·7%), while 0·5% reported fourth-level education. Most participants lived in urban areas (79·0%). Regarding geographic region, 45·3% resided in the Andean highlands (Sierra), 35·4% in the Coastal region (Costa), and 19·4% in the Amazon region (Amazonian). Most respondents self-identified as Mestizo (87·9%), while 4·7% identified as Indigenous. Because waiting time was right-skewed, it was summarized using medians and interquartile ranges; the overall median waiting time was 30 days (IQR 15–45). Median waiting time was 30 days (IQR 15–45) among participants reporting standardized access and 25 days (IQR 12–40) among those reporting non-standardized access. Participant characteristics and waiting times by access pathway are shown in Table 1.

**Table 1:** Sociodemographic characteristics and waiting times of study participants, by access pathway. Ecuador, 2025.

Variable	Total (n = 4436)	Standardized (n = 1431)	Non-standardized (n = 3005)
Age, mean ± SD	43·22 ± 15·38	43·93 ± 16·32	42·89 ± 14·90
Sex: Female	2818 (63·5)	951 (66·5)	1867 (62·1)
Sex: Male	1618 (36·5)	480 (33·5)	1138 (37·9)
Education: None	150 (3·4)	52 (3·6)	98 (3·3)
Education: Primary	1195 (26·9)	365 (25·5)	830 (27·6)
Education: Secondary	2286 (51·5)	733 (51·2)	1553 (51·7)
Education: Third level	785 (17·7)	270 (18·9)	515 (17·1)
Education: Fourth level	20 (0·5)	11 (0·8)	9 (0·3)
Area of residence: Urban	3503 (79·0)	1079 (75·4)	2424 (80·7)
Area of residence: Rural	933 (21·0)	352 (24·6)	581 (19·3)
Geographic region: Andean highlands	2008 (45·3)	654 (45·7)	1354 (45·1)
Geographic region: Coastal	1569 (35·4)	532 (37·2)	1037 (34·5)
Geographic region: Amazon	859 (19·4)	245 (17·1)	614 (20·4)
Ethnicity: Mestizo	3901 (87·9)	1238 (86·5)	2663 (88·6)
Ethnicity: Indigenous	209 (4·7)	81 (5·7)	128 (4·3)
Ethnicity: Afro-Ecuadorian	161 (3·6)	71 (5·0)	90 (3·0)
Ethnicity: Montubio	103 (2·3)	19 (1·3)	84 (2·8)
Ethnicity: White	62 (1·4)	22 (1·5)	40 (1·3)
Household income quintile: Q1	888 (20·0)	229 (16·0)	659 (21·9)
Household income quintile: Q2	887 (20·0)	244 (17·1)	643 (21·4)
Household income quintile: Q3	887 (20·0)	280 (19·6)	607 (20·2)
Household income quintile: Q4	887 (20·0)	393 (27·5)	494 (16·4)
Household income quintile: Q5	887 (20·0)	285 (19·9)	602 (20·0)
Waiting time (days), median (IQR)	30·0 (15·0–45·0)	30·0 (15·0–45·0)	25·0 (12·0–40·0)

**Fig 1:**
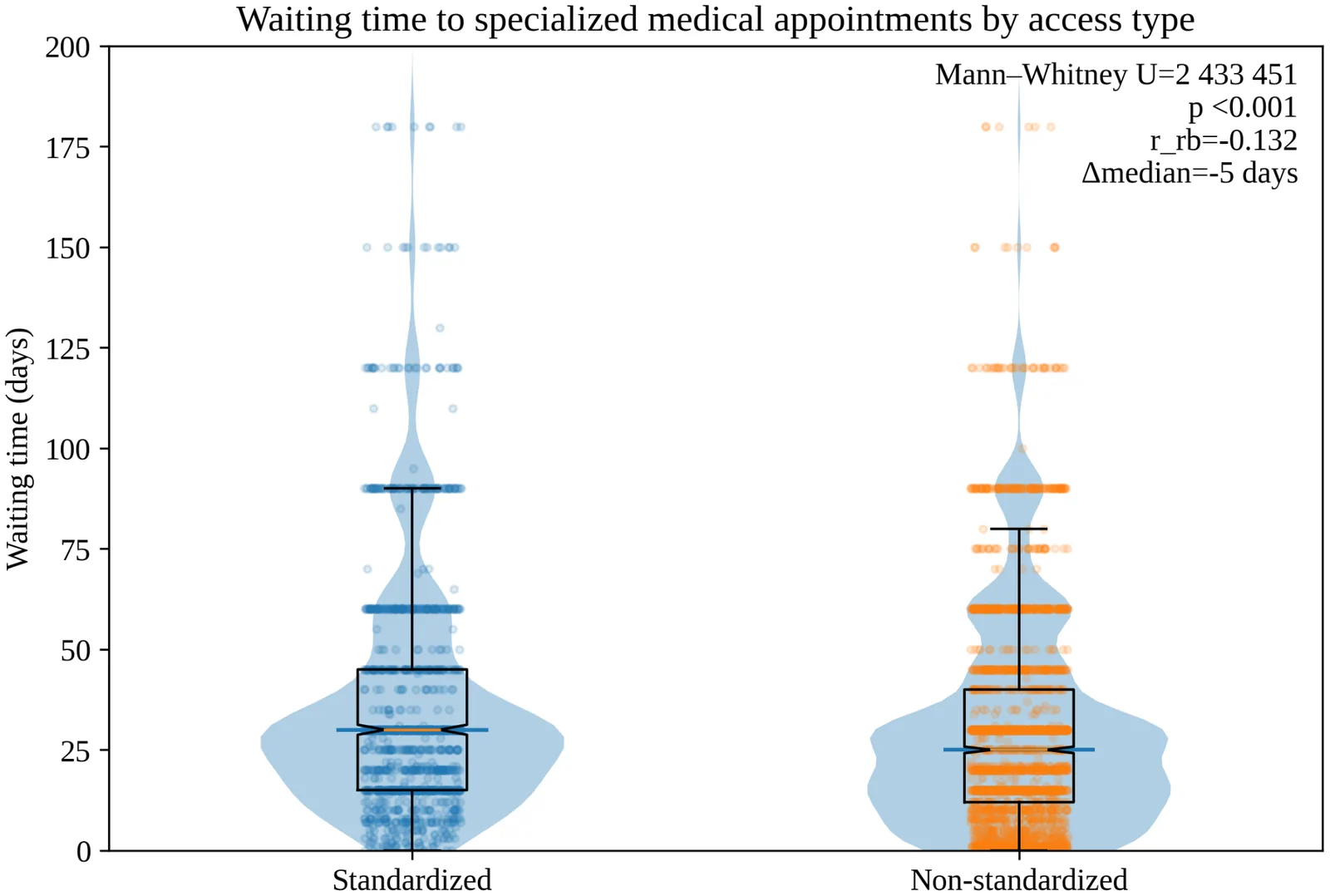
Waiting time for specialist appointments by access type (standard vs non-standard).

Waiting time to specialist appointments differed by access type. As shown in Fig 1, the distribution of waiting time was right skewed in both groups; therefore, results were summarized using medians and interquartile ranges. Participants with standardized access had a median waiting time of 30 days (IQR 15–45), whereas those reporting non-standardized access had a median waiting time of 25 days (IQR 12–40). The difference was statistically significant (Mann–Whitney U = 2,433,451; p < 0·001), with a small effect size (rank-biserial r = −0·132), indicating shorter waiting times among participants who reported non-standardized access. For visualization purposes, the y-axis was truncated at 200 days; three observations above this threshold were not displayed, without affecting the statistical comparison, which was conducted using the full dataset.

Fig 1 shows the distribution of waiting time in days. The inset shows the results of the Mann-Whitney U test and the rank-biserial effect size. For ease of visualization, the y-axis was truncated at 200 days; the three observations exceeding this threshold are not shown.

After accounting for clustering by hospital, crude associations were imprecise for most sociodemographic variables; fourth-level education and household income quintile Q4 were associated with lower odds of non-standardized access (Table 2). In the multivariable model adjusting for sex, age, education, geographic region, ethnicity, household income quintile, and area of residence, female participants had lower odds of non-standardized access than male participants (aOR 0.79, 95% CI 0.63–1.00; p = 0.048), although the statistical evidence was borderline after accounting for clustering. Rural residents also had lower odds than urban residents (aOR 0.67, 95% CI 0.49–0.93). Participants with fourth-level education had lower odds than those with secondary education (aOR 0.41, 95% CI 0.22–0.77), and participants in Q4 had lower odds than those in the highest income quintile (aOR 0.61, 95% CI 0.45–0.85). Age, geographic region, ethnicity, and the remaining education and income categories showed no clear evidence of association.

**Table 2:** Crude and adjusted odds ratios for non-standardized access to specialist appointments. Ecuador, 2025.

Variable	Crude OR (95% CI)	Adjusted OR (95% CI)
Age, per year	1.00 (0.99–1.00)	0.99 (0.98–1.00)
Sex: Male (reference)	1.00 (ref)	1.00 (ref)
Sex: Female	0.83 (0.66–1.04)	0.79 (0.63–1.00)
Education: Secondary (reference)	1.00 (ref)	1.00 (ref)
Education: None	0.89 (0.53–1.49)	0.90 (0.53–1.53)
Education: Primary	1.07 (0.88–1.31)	1.02 (0.86–1.21)
Education: Third level	0.90 (0.72–1.12)	0.95 (0.76–1.18)
Education: Fourth level	0.39 (0.22–0.69)	0.41 (0.22–0.77)
Region: Sierra (reference)	1.00 (ref)	1.00 (ref)
Region: Costa	0.94 (0.63–1.40)	0.89 (0.61–1.31)
Region: Amazonian	1.21 (0.71–2.06)	1.21 (0.72–2.02)
Ethnicity: Mestizo (reference)	1.00 (ref)	1.00 (ref)
Ethnicity: Indigenous	0.73 (0.36–1.52)	0.85 (0.38–1.89)
Ethnicity: Other	0.89 (0.30–2.61)	1.00 (0.33–2.99)
Income: Q5 (reference)	1.00 (ref)	1.00 (ref)
Income: Q1	1.36 (0.66–2.82)	1.55 (0.74–3.27)
Income: Q2	1.25 (0.65–2.38)	1.31 (0.68–2.49)
Income: Q3	1.03 (0.59–1.78)	1.08 (0.63–1.85)
Income: Q4	0.60 (0.43–0.82)	0.61 (0.45–0.85)
Residence: Urban (reference)	1.00 (ref)	1.00 (ref)
Residence: Rural	0.73 (0.51–1.06)	0.67 (0.49–0.93)

Note: OR, odds ratio; CI, confidence interval. All 95% CIs were calculated using standard errors clustered at the hospital level, with a CR1 finite-sample correction and t-distribution inference with 25 degrees of freedom (26 hospitals). Other ethnicity includes Afro-Ecuadorian, Montubio, and White participants.

In a median regression model adjusted for age, sex, education, geographic region, ethnicity, household income quintile, area of residence, specialty, and hospital, and using hospital-clustered inference, non-standardized access was associated with a shorter conditional median waiting time than standardized access (adjusted median difference −3.6 days, 95% CI −6.2 to −1.0; p = 0.008).

Descriptively, among direct-access specialties, the median waiting time was 30 days (IQR 15–30) for standardized access and 15 days (IQR 5–30) for non-standardized access. Among referral-required specialties, the corresponding medians were 30 days (IQR 15–45) and 25 days (IQR 15–45), respectively (Table 3). In the adjusted interaction model, non-standardized access was associated with a 7.9-day shorter conditional median waiting time in direct-access specialties (adjusted median difference −7.9 days, 95% CI −13.7 to −2.1; p = 0.010) and a 3.1-day shorter conditional median waiting time in referral-required specialties (adjusted median difference −3.1 days, 95% CI −5.9 to −0.2; p = 0.036). However, the interaction between access pathway and referral-requirement stratum was not statistically significant (interaction estimate −4.8 days, 95% CI −11.2 to 1.6; p = 0.136), providing no clear evidence that the association differed between the two strata.

**Table 3:** Waiting time to specialist appointments by access pathway and specialty referral requirement, with adjusted conditional median differences. Ecuador, 2025.

Specialty stratum	Standard access, n	Standard waiting time, median (IQR), days	Non-standard access, n	Non-standard waiting time, median (IQR), days	Adjusted conditional median difference (non-standardized minus standardized), days (95% CI)†
Direct-access specialties (Dentistry, General Medicine, Family Medicine and Psychology) *	130	30 (15–30)	281	15 (5–30)	−7·9 (−13.7 to −2·1)
**Referral-required specialties (all other specialties)**	1301	30 (15–45)	2724	25 (15–45)	−3·1 (−5.9 to −0.2)

* The direct-access specialties were defined using the specialty codes Dentistry, General Medicine, Family Medicine and Psychology.

†Adjusted conditional median differences were estimated from a single median regression model containing sociodemographic covariates, hospital and individual-specialty fixed effects, and the interaction between access pathway and referral-requirement stratum, with hospital-clustered inference. The interaction estimate was −4.8 days (95% CI −11.2 to 1.6; p = 0.136).

## Discussion

This study found that, among adults who attended a scheduled outpatient specialist consultation in Ecuadorian public hospitals, non-standardized access was associated with a 3.6-day shorter conditional median waiting time after adjustment for sociodemographic characteristics, specialty, and hospital, with hospital-clustered inference. Although the adjusted association was numerically larger in direct-access specialties, the formal interaction test did not provide clear evidence that the association differed according to specialty referral requirement. The stratum-specific estimates should therefore be interpreted as adjusted contrasts within each group rather than as conclusive evidence of effect modification. Taken together, these findings suggest that, within routine outpatient specialist care, parallel access pathways may shape the timeliness of care.

Existing evidence suggests that waiting time is a common non-price rationing mechanism in publicly financed systems, but it is not necessarily experienced equally across social groups [3,4,7]. Studies using survey data from European settings have shown that higher socioeconomic position, particularly education, can be associated with shorter waits for specialist consultation, challenging the assumption that waiting lists are inherently equitable [4,6]. More recent cross-country analyses likewise indicate that socioeconomic gradients in waiting time can persist even in systems designed to provide universal coverage, reflecting differences in patients’ ability to navigate care pathways and in how resources and capacity constraints are managed [3,13]. Although much of this literature comes from OECD contexts, qualitative and mixed evidence from Latin America has highlighted that long waiting times after referral are a recurrent organizational barrier within public care networks, often interacting with geographic access barriers and unequal support networks [8,14].

Our findings complement this literature by explicitly quantifying a parallel phenomenon that is frequently discussed but little measured in individual-level studies: the coexistence of standardized and non-standardized access pathways to specialist care, and their relationship to waiting time. Evidence from a recent systematic review on self-referral and direct-access pathways suggests that alternative access routes can have mixed effects but may frequently widen inequalities, because individuals with greater resources or social advantage are more likely to use them effectively [15]. In this context, the shorter waiting times observed among those using non-standardized access are consistent with the idea that non-standardized pathways may bypass bottlenecks in standard scheduling processes, but they also raise concerns that timeliness may depend partly on social connections rather than clinical need, particularly in settings where institutional capacity and referral governance are constrained.

Several mechanisms could plausibly explain why non-standardized access was associated with shorter waiting times in our context. First, non-standardized access may reflect the mobilization of “connection” social capital, whereby personal links with individuals with institutional influence provide privileged information (e.g., on appointment availability) or facilitate overcoming administrative hurdles that would otherwise slow down the standard process [16]. In publicly funded systems with limited specialist capacity, these advantages may translate into earlier appointment allocation without necessarily implying a change in the underlying clinical need.

Second, the observed pattern is compatible with bypassing behaviour documented in other health systems, in which patients do not follow the intended gatekeeping sequence and instead seek direct entry to higher-level care, often influenced by perceived quality, convenience, or prior experience with primary care and its coordination function [17]. Even where standard rules exist, weak enforcement or parallel channels can enable direct entry, particularly when patients perceive the standard route as slow or uncertain.

Third, specialty referral requirements provide useful context for interpreting the association between access pathway and waiting time. The adjusted point estimate was numerically larger in direct-access specialties, but the formal interaction test did not establish effect modification by referral requirement. Institutional referral rules should therefore be considered plausible contextual factors rather than demonstrated modifiers of the association. Within referral-required specialties, non-standardized access was also associated with a shorter adjusted conditional median waiting time. Referral pathways nevertheless remain clinically important because they are intended to direct patients from primary care to higher-complexity services when specialist assessment is required [11]. Because we did not measure clinical appropriateness or perceived urgency, we cannot determine whether faster non-standardized access reflected bypassing of clinically necessary coordination or attempts to overcome administrative and geographic barriers [16].

The distribution of access pathways and waiting times in our study also points to sociodemographic heterogeneity relevant to equity in the Ecuadorian public system. After accounting for hospital clustering, lower odds of non-standardized access were observed among female participants, rural residents, participants with fourth-level education, and those in household income quintile Q4, whereas geographic region and the remaining income categories showed no clear associations. Previous Latin American evidence has documented socioeconomic gradients in health-system coverage and quality [18]. However, our estimates did not show a simple monotonic socioeconomic gradient, suggesting that non-standardized pathways may reflect heterogeneous coping strategies rather than being used exclusively by socially advantaged groups.

The lower propensity to use non-standardized access among rural residents in our sample may be explained by structural constraints in rural settings, including fewer specialist resources, longer travel distances, and weaker institutional connectivity. These factors can reduce opportunities to activate influential networks within the health system. Evidence from other contexts indicates that rural populations often face systematic barriers to specialty care, including provider scarcity and longer travel distances, which can compound disadvantages even when standard coverage exists [19]. Our regional differences further support a territorial interpretation: where specialist supply, administrative capacity, and interfacility referral connectivity differ by macro-region, non-standardized access may emerge differentially as either a substitute for, or a bypass of, the standard route.

The socioeconomic patterning we observed also resonates with the longstanding debate that waiting lists, although often framed as an equitable rationing mechanism, can generate unequal non-monetary prices when individuals differ in their ability to navigate the system, mobilize information, or secure timely appointments through non-standardized channels [4,6]. In this sense, non-standardized access can be understood as one manifestation of social capital in health-care utilization: social ties and perceived support may facilitate access to services, but their distribution can also reproduce inequities if the ability to leverage such ties is socially patterned. Notably, the presence of heterogeneity in both access pathways and waiting times strengthens the policy relevance of our findings, because it suggests that reforms aimed solely at reducing average waiting time may be insufficient unless they also address the mechanisms through which some groups can consistently obtain faster access than others.

Our analytic strategy was designed to align with the distributional characteristics of waiting time and to provide estimates that are interpretable for policy. Waiting time for specialist appointments was strongly right-skewed, so we first used a non-parametric comparison to assess whether the overall distributions differed between standardized and non-standardized access, and we presented this comparison visually to avoid overreliance on a single summary statistic. We also reported a rank-based effect size to complement the hypothesis test.

This study has several limitations. First, the analytic sample was conditioned on successful attendance at a scheduled outpatient specialist consultation. We therefore did not observe individuals who sought specialist care but did not obtain an appointment, abandoned the process before attendance, did not attend the scheduled consultation, or sought care outside the public system. Restricting the analysis to successful attendees may have introduced selection bias if the probability of completing the care-seeking process was related to both access pathway and waiting time or to factors associated with them, such as socioeconomic resources, clinical need, or persistence in navigating the system. Because patients experiencing the longest waits may have abandoned the process before attendance, our observed waiting-time difference likely represents a conservative lower bound of the true difference among all patients who initially sought specialist care. This interpretation is especially plausible if dropout was more frequent among patients experiencing long waits through standardized pathways. Nevertheless, because unsuccessful attempts were not observed, the precise magnitude of this selection bias cannot be quantified from the available data. Accordingly, our findings apply to adults who successfully attended scheduled specialist consultations in participating public hospitals and should not be generalized to all individuals seeking specialist care.

Second, waiting time and access pathway were self-reported and may have been affected by recall error or under-reporting, particularly for non-standardized access. Third, although emergency care and unscheduled urgent visits were excluded by design, we did not directly measure clinical priority among scheduled outpatient consultations; residual confounding by consultation priority therefore remains possible. Fourth, the cross-sectional design limits causal interpretation. Fifth, the study was hospital-based and restricted to participating public hospitals, which may limit generalizability to other public hospitals, private facilities, or individuals who never reached hospital-based specialist care. Finally, scheduling practices, specialist availability, and administrative procedures may vary across hospitals. We included hospital fixed effects and hospital-clustered inference to account for measured and unmeasured time-invariant differences between hospitals and for within-hospital dependence; however, residual confounding by unmeasured differences in capacity, referral implementation, and scheduling policies may remain.

These findings suggest that reducing waiting times in outpatient specialist care may require not only additional specialist capacity but also greater transparency in how appointments are allocated. Among patients who successfully reached specialist care, shorter waiting times through non-standardized access pathways indicate that timeliness may depend partly on factors outside the formal scheduling process. The association was observed in both referral-requirement strata, but the formal interaction test did not provide clear evidence that its magnitude differed according to specialty referral requirement. Future studies should use longitudinal referral and scheduling records to follow patients from referral initiation through appointment completion and capture unmet demand, unsuccessful attempts to obtain appointments, non-attendance, abandonment of the care-seeking process, and movement between public and private care.
