# Supplementary material for: Waiting time for scheduled outpatient specialist consultations by access pathway in public hospitals in Ecuador
Source: PLoS One. 2026 Sep 17;21(9):e0349550. doi: 10.1371/journal.pone.0349550 (PMC13585269; doi:10.1371/journal.pone.0349550)
Supplement: Supplementary file 2 [file pone.0349550.s005.pdf]

Response to the Academic Editor and Reviewers

Manuscript ID: PONE-D-26-21785R1

Title: Waiting time for scheduled outpatient specialist consultations by access pathway in public hospitals in Ecuador

Dear Dr. Zarei and Reviewers,

We sincerely thank the Academic Editor and both reviewers for their careful evaluation of our revised manuscript. We are grateful for the reviewers' confirmation that the previous comments were adequately addressed and that the manuscript is suitable for publication. In response to the Academic Editor's additional methodological concerns, we conducted the requested reanalyses and revised the Abstract, Methods, Results, Tables 2 and 3, Discussion, and Conclusion. Below, we provide a point-by-point response.

In addition, because the analyses were re-run using the dataset corresponding to the final revised analyses, we updated the Zenodo deposit and the Data Availability Statement. The anonymized minimal dataset underlying the findings of this study, together with the data dictionary, is publicly available in Zenodo at https://doi.org/10.5281/zenodo.22166322.

Response to the Academic Editor

Comment 1: Hospital clustering

"Your data are clustered by hospital. Patients within the same hospital are more similar to each other than to patients in other hospitals due to shared scheduling practices, specialist availability, and administrative procedures. Your current models do not account for this clustering, which violates the independence assumption of your regression models. This can lead to incorrect standard errors and invalid inference.

Recommendation: Please reanalyse all regression models accounting for hospital clustering."

Response: We thank the Academic Editor for identifying this important issue. We reanalysed all regression models using hospital-clustered sandwich variance estimators. This correction was applied to the crude and adjusted logistic regressions, the primary median regression, and the interaction median regression. All clustered models used 26 hospital clusters, a CR1 finite-sample correction, and confidence intervals and hypothesis tests based on a t distribution with 25 degrees of freedom.

The Statistical analysis subsection has been revised to describe the hospital-clustered inference. Table 2 and the corresponding Results paragraph have also been updated with hospital-clustered confidence intervals. The logistic-regression point estimates remained unchanged, but several confidence intervals widened after accounting for within-hospital dependence. In particular, age, Amazonian region, and household income quintiles Q1 and Q2 no longer showed clear adjusted associations with non-standardized access. The associations for rural residence, fourth-level education, and household income quintile Q4 remained statistically supported, while the association for female sex was borderline after clustering.

During this reanalysis, we also identified and corrected a transcription inconsistency in the previously reported crude odds ratio for household income quintile Q1. The crude odds ratio was corrected from 1.61 to 1.36; the adjusted odds ratio remained unchanged at 1.55.

The revised Methods text states:

"Because participants were clustered within hospitals, all regression models used hospital-clustered sandwich variance estimators. We applied a CR1 finite-sample correction and based

confidence intervals and hypothesis tests on a t distribution with 25 degrees of freedom, corresponding to 26 hospital clusters. For the median-regression models, the clustered sandwich covariance matrix was based on quantile scores and a residual-density estimate."

Comment 2: Specialty and hospital as covariates

"Your primary waiting-time model does not include specialty or hospital as covariates. Both are important potential confounders. Specialty affects waiting times and referral patterns, and hospital affects capacity and scheduling policies. These factors could bias your estimates.

Recommendation: Please adjust your primary model for both specialty and hospital, or provide a strong justification for not doing so."

Response: We agree with the Academic Editor and have adjusted the primary median regression model for both individual specialty and hospital. The revised model included age, sex, education, geographic region, ethnicity, household income quintile, area of residence, individual-specialty fixed effects, and hospital fixed effects. Hospital-clustered inference was applied as described above. All 4,436 participants were retained in the final model.

After this adjustment, non-standardized access was associated with a 3.6-day shorter conditional median waiting time compared with standardized access (adjusted median difference -3.6 days, 95% CI -6.2 to -1.0; p = 0.008). The magnitude was similar to the previously reported estimate of -3.2 days, although the confidence interval was wider after accounting for specialty, hospital, and within-hospital dependence.

The Abstract, Statistical analysis subsection, Results, and opening paragraph of the Discussion have been updated accordingly. The revised Results text states:

"In a median regression model adjusted for age, sex, education, geographic region, ethnicity, household income quintile, area of residence, specialty, and hospital, and using hospital-clustered inference, non-standardized access was associated with a shorter conditional median waiting time than standardized access (adjusted median difference -3.6 days, 95% CI -6.2 to -1.0; p = 0.008)."

Comment 3: Interaction test

"You stratified the analysis by referral requirement and concluded that the effect of access pathway is larger in direct-access specialties. However, comparing estimates from two separate stratified models is not sufficient to conclude that the effect differs between groups.

Recommendation: Please formally test whether the effect of access pathway differs by referral requirement. Fit a single model that allows you to test this interaction, and report the result."

Response: We thank the Academic Editor for this clarification. We fitted a single median regression model containing an interaction between access pathway and specialty referral-requirement stratum. The model included the same sociodemographic covariates, individual-specialty fixed effects, hospital fixed effects, and hospital-clustered inference as the primary model. Because referral requirement was determined by individual specialty, the referral-requirement main effect was absorbed by the individual-specialty fixed effects, while the interaction term remained identifiable and was formally tested.

In the adjusted interaction model, non-standardized access was associated with a 7.9-day shorter conditional median waiting time in direct-access specialties (adjusted median difference -7.9 days, 95% CI -13.7 to -2.1; p = 0.010) and a 3.1-day shorter conditional median waiting time in referral-required specialties (adjusted median difference -3.1 days, 95% CI -5.9 to -0.2; p =

0.036). However, the interaction term was not statistically significant (interaction estimate -4.8 days, 95% CI -11.2 to 1.6; p = 0.136).

We therefore revised the Abstract, Methods, Results, Table 3, Discussion, and Conclusion to avoid concluding that the association was statistically different between the two referral-requirement strata. The revised manuscript now states that the adjusted point estimate was numerically larger in direct-access specialties, but that the formal interaction test did not provide clear evidence of effect modification by referral requirement.

The revised Results text states:

"However, the interaction between access pathway and referral-requirement stratum was not statistically significant (interaction estimate -4.8 days, 95% CI -11.2 to 1.6; p = 0.136), providing no clear evidence that the association differed between the two strata."

Comment 4: Selection bias — Discussion

"You correctly acknowledge in the limitations that your sample only includes patients who successfully attended a scheduled consultation. However, you do not discuss the implications of this bias for your interpretation.

Recommendation: In the Discussion section, please add a paragraph explaining that:

Your estimates represent a lower bound of the true waiting time difference, because patients with the longest waits may have dropped out before attending.

Your findings only generalize to patients who successfully navigated the system to attendance, not to those who never obtained an appointment.

Future studies should capture unsuccessful attempts to access care."

Response: We agree with the Academic Editor and have substantially expanded the discussion of selection bias in the Limitations subsection. The revised text explains that the analytic sample was conditioned on successful attendance and did not include individuals who failed to obtain an appointment, abandoned the process, did not attend the scheduled consultation, or sought care outside the public system.

We now explicitly state that, because patients experiencing the longest waits may have abandoned the process before attendance, the observed waiting-time difference likely represents a conservative lower bound of the true difference among all individuals who initially sought specialist care. We also clarify that the findings apply to adults who successfully attended scheduled specialist consultations in participating public hospitals and should not be generalized to all individuals seeking specialist care.

Finally, the Conclusion now recommends that future studies use longitudinal referral and scheduling records to follow patients from referral initiation through appointment completion and capture unmet demand, unsuccessful attempts to obtain appointments, non-attendance, abandonment, and movement between public and private care.

The revised Limitations text includes the following statement:

"Because patients experiencing the longest waits may have abandoned the process before attendance, our observed waiting-time difference likely represents a conservative lower bound of the true difference among all patients who initially sought specialist care. This interpretation is especially plausible if dropout was more frequent among patients experiencing long waits through standardized pathways. Nevertheless, because unsuccessful attempts were not observed, the precise magnitude of this selection bias cannot be quantified from the available data."

We also updated the remaining limitations to reflect the new analyses. Specifically, we removed the previous statement that hospital-level heterogeneity had not been modelled and now acknowledge that, although hospital fixed effects and hospital-clustered inference were used, residual confounding by unmeasured differences in capacity, referral implementation, and scheduling policies may remain. We also clarified that all 4,436 participants had complete data for the variables included in the final regression models.

Response to Reviewer #1

Reviewer #1 confirmed that all comments from the previous review round had been adequately addressed and that the manuscript was suitable for publication.

Response: We sincerely thank Reviewer #1 for the careful re-evaluation of our manuscript and for confirming that the previous comments were adequately addressed. We appreciate the reviewer's positive assessment and recommendation for publication. No additional changes were requested by Reviewer #1.

Response to Reviewer #2

Reviewer #2 confirmed that the manuscript met the criteria for PLOS ONE and recommended publication.

Response: We sincerely thank Reviewer #2 for the positive evaluation and recommendation for publication. No additional changes were requested by Reviewer #2.

We again thank the Academic Editor and reviewers for their constructive comments. We believe that the requested reanalyses and corresponding revisions have strengthened the methodological rigor, transparency, and interpretation of the manuscript.

Sincerely,

Marcelo Armijos Briones
On behalf of all authors
