# Supplementary material for: Waiting time for scheduled outpatient specialist consultations by access pathway in public hospitals in Ecuador
Source: PLoS One. 2026 Sep 17;21(9):e0349550. doi: 10.1371/journal.pone.0349550 (PMC13585269; doi:10.1371/journal.pone.0349550)
Supplement: Supplementary file 3 [file pone.0349550.s001.docx]

**General Overview**

The manuscript investigates a critical and often overlooked aspect of health equity: how different access pathways (standardized vs. non-standardized/informal) influence waiting times for specialist care in a public hospital system. Using a large sample (n = 4,436), the authors provide robust empirical evidence that informal pathways systematically result in shorter wait times, particularly for direct-access specialties. This study is highly relevant for policymakers in Latin America and other regions facing similar health system fragmentation.

**Specific Comments by Section**

1. Title and Abstract (Pages 1–3)

Title (Page 1): The title is descriptive and accurately reflects the study's scope.

Abstract (Pages 2–3, Lines 40–71): The abstract provides a comprehensive summary of the objective, methods, and key findings.

Comment: The abstract clearly highlights the adjusted median difference of -3.2 days and the significant disparity in direct-access specialties (-15.0 days). This clarity is a major strength.

2. Introduction (Pages 3–5, Lines 77–121)

Contextualization (Pages 3–4): The authors effectively frame waiting time as a non-monetary rationing mechanism and establish that delays can lead to disease progression.

Conceptual Framework (Pages 4–5): The introduction of "social connections" as a determinant of timeliness is well-supported by the cited literature regarding Latin American health system fragmentation.

Recommendation: On Page 4 (Line 94), the authors mention that patient-level evidence remains limited in the region. This study successfully bridges that gap.

3. Methods (Pages 6–9, Lines 122–208)

Design and Setting (Page 6): The study uses a cross-sectional hospital survey conducted between July and October 2024.

Definitions (Pages 7–8):

Standardized access is clearly defined as the institutional referral pathway.

Non-standardized access includes informal routes through friends, family, or other personal networks.

Statistical Analysis (Pages 8–9): The use of the Mann–Whitney U test for skewed distributions and quantile regression (τ = 0.50) for adjusted median differences is statistically sound for this type of data.

Ethics (Page 9): The study received appropriate ethics committee approval (CEISH-ITSUP code 1718079732) and obtained informed consent.

4. Results (Pages 10–14, Lines 210–284)

Demographics (Pages 10–11): Table 1 shows that women accounted for 63.5% of the sample and the majority identified as Mestizo.

Key Findings (Page 11, Lines 231–234): The overall median waiting time was 30 days for standardized access versus 25 days for non-standardized access, a statistically significant difference (p < 0.001).

Stratification (Pages 13–14, Lines 268–277): Table 3 presents the most striking finding: in direct-access specialties (e.g., Dentistry, Psychology), the median difference was -15.0 days.

Comment: The presentation of Figure 1 (violin plot) effectively visualizes the right-skewed distribution of waiting times across both groups.

5. Discussion (Pages 15–20, Lines 286–417)

Interpretation (Pages 15–16): The authors correctly identify that non-standardized pathways may reflect the mobilization of "connection" social capital to bypass administrative hurdles.

Equity Implications (Page 17): The discussion highlights a major risk: patients following formal routes (who were referred by a physician) may wait longer than those using informal routes based on self-perceived (and potentially inaccurate) needs.

Limitations (Pages 19–20, Lines 396–408): The manuscript appropriately addresses limitations, including recall bias, the cross-sectional design, and the exclusion of patients who never successfully obtained an appointment.

6. Conclusion (Page 20, Lines 410–417)

Final Summary: The conclusion is supported by the data, stating that timeliness depends partly on factors outside the formal scheduling process.

Recommendation: The call for greater transparency in appointment allocation is a logical and actionable policy recommendation based on these findings.

**Reviewer Recommendation**

**Verdict: Accept with Minor Revisions.**

**Minor Points for the Authors:**

Data Availability (Page 7): While a Google Drive link is provided, PLOS ONE typically requires data to be in a permanent repository (like Figshare or Dryad) unless there are specific privacy concerns. The authors should confirm if this link is permanent.

Terminology (Page 3, Line 51): Ensure that "non-system-based" is used consistently to describe non-standardized pathways to avoid confusion.

Clarity (Page 14, Line 281): In Table 3, the note defining direct-access specialties includes "Family Medicine," which is an important detail for understanding the Ecuadorian context
