# Supplementary material for: Waiting time for scheduled outpatient specialist consultations by access pathway in public hospitals in Ecuador
Source: PLoS One. 2026 Sep 17;21(9):e0349550. doi: 10.1371/journal.pone.0349550 (PMC13585269; doi:10.1371/journal.pone.0349550)
Supplement: Supplementary file 4 [file pone.0349550.s004.docx]

**Manuscript ID:** PONE-D-26-21785
**Title:** Waiting time for scheduled outpatient specialist consultations by access pathway in public hospitals in Ecuador

Dear Dr. Machado and Reviewers,

We sincerely thank the Academic Editor and both reviewers for their careful evaluation of our manuscript and for the constructive comments. We are grateful for the positive assessment of the technical soundness, statistical analysis, clarity, and relevance of the study. We have revised the manuscript and the submission information according to the editorial requirements and reviewers’ comments. Below, we provide a point-by-point response to each issue raised.

**Response to the Academic Editor and Journal Requirements**

**Editor’s requirement 1:** Please ensure that your manuscript meets PLOS ONE’s style requirements, including those for file naming.

**Response:** We thank the Academic Editor for this reminder. We have reviewed the revised files according to PLOS ONE’s formatting and file-naming requirements. We are submitting the required files separately: a response letter, a revised manuscript with tracked changes, and a clean revised manuscript.

**Editor’s requirement 2:** Please note that funding information should not appear in any section or other areas of your manuscript. We will only publish funding information present in the Funding Statement section of the online submission form. Please remove any funding-related text from the manuscript.

**Response:** We thank the Academic Editor for this clarification. We have removed the funding-related text from the manuscript. Specifically, the previous “Funding” section was deleted from the revised manuscript. The Funding Statement will be provided only in the corresponding section of the online submission form and in the cover letter, as requested.

The updated Funding Statement is as follows:

**Updated Funding Statement:**
The authors received no specific funding for this work. Pvalor SAS provided support in the form of salary for author NAB, but did not have any additional role in the study design, data collection and analysis, decision to publish, or preparation of the manuscript. The specific role of this author is articulated in the author contributions section.

**Editor’s requirement 3:** Please provide an amended Funding Statement declaring the commercial affiliation, as well as a statement regarding the Role of Funders in your study. Please also provide an updated Competing Interests Statement declaring this commercial affiliation.

**Response:** We thank the Academic Editor for this request. We have updated the Funding Statement as indicated above. We have also updated the Competing Interests Statement to declare the commercial affiliation of author NAB with Pvalor SAS and to confirm that this affiliation does not alter our adherence to PLOS ONE policies on sharing data and materials.

**Updated Competing Interests Statement:**
I have read the journal’s policy and the authors of this manuscript have the following competing interests: Nicole Armijos Bazurto is employed by Pvalor SAS, Portoviejo, Ecuador. This commercial affiliation did not influence the study design, data collection and analysis, decision to publish, or preparation of the manuscript. This does not alter our adherence to PLOS ONE policies on sharing data and materials. The remaining authors declare no competing interests.

**Editor’s requirement 4:** We note you have included a table to which you do not refer in the text of your manuscript. Please ensure that you refer to Table 1 in your text; if accepted, production will need this reference to link the reader to the Table.

**Response:** We thank the Academic Editor for identifying this issue. We have added an explicit reference to Table 1 in the Results section, immediately before the table. The revised text now reads:

“Participant characteristics and waiting times by access pathway are shown in Table 1.”

**Response to Reviewer #1**

We thank Reviewer #1 for the careful and positive evaluation of our manuscript and for recognizing the relevance of this study for health equity and health system policy in Latin America.

**Reviewer #1, General Overview:** The manuscript investigates a critical and often overlooked aspect of health equity: how different access pathways influence waiting times for specialist care in a public hospital system. Using a large sample, the authors provide robust empirical evidence that informal pathways systematically result in shorter wait times, particularly for direct-access specialties. This study is highly relevant for policymakers in Latin America and other regions facing similar health system fragmentation.

**Response:** We thank the reviewer for this positive assessment. We appreciate the recognition of the relevance of the study and its contribution to the literature on waiting times, access pathways, and health equity in public hospital systems.

**Reviewer #1, Title and Abstract:** The title is descriptive and accurately reflects the study’s scope. The abstract provides a comprehensive summary of the objective, methods, and key findings. The abstract clearly highlights the adjusted median difference of −3.2 days and the significant disparity in direct-access specialties.

**Response:** We thank the reviewer for this positive comment. No changes were required in the title or abstract.

**Reviewer #1, Introduction:** The authors effectively frame waiting time as a non-monetary rationing mechanism and establish that delays can lead to disease progression. The introduction of “social connections” as a determinant of timeliness is well supported by the cited literature. The study successfully bridges the gap in patient-level evidence in the region.

**Response:** We thank the reviewer for this assessment. No substantive changes were required in the Introduction. However, as part of the minor typographical revision, we corrected the phrase “An classic work” to “A classic work.”

**Reviewer #1, Methods:** Standardized access is clearly defined as the institutional referral pathway. Non-standardized access includes informal routes through friends, family, or other personal networks. The use of the Mann–Whitney U test and quantile regression is statistically sound. The study received appropriate ethics committee approval and obtained informed consent.

**Response:** We thank the reviewer for this positive assessment of the methodological and statistical approach. To further improve clarity and address the reviewer’s terminology comment below, we revised the exposure definition to explicitly state that non-standardized access refers to informal or non-system-based mechanisms. The revised text in the Methods section now reads:

“Non-standardized access was defined as obtaining the appointment through informal or non-system-based mechanisms, including through a friend or family member or through a person who was not a friend or family member. Throughout the manuscript, non-standardized access refers to these informal or non-system-based mechanisms.”

**Reviewer #1, Results:** Table 1 shows the demographic characteristics of the sample. The overall median waiting time was 30 days for standardized access versus 25 days for non-standardized access. Table 3 presents the most striking finding: in direct-access specialties, the median difference was −15.0 days. Figure 1 effectively visualizes the right-skewed distribution of waiting times.

**Response:** We thank the reviewer for this positive comment. In response to the Academic Editor’s request, we added a direct reference to Table 1 in the Results section. We also revised the description of direct-access specialties in the Results section to include Family Medicine consistently.

**Reviewer #1, Discussion:** The authors correctly identify that non-standardized pathways may reflect the mobilization of “connection” social capital to bypass administrative hurdles. The discussion highlights a major risk: patients following formal routes may wait longer than those using informal routes based on self-perceived needs. The limitations are appropriately addressed.

**Response:** We thank the reviewer for this assessment. No substantive changes were required in the Discussion.

**Reviewer #1, Conclusion:** The conclusion is supported by the data, stating that timeliness depends partly on factors outside the formal scheduling process. The call for greater transparency in appointment allocation is a logical and actionable policy recommendation.

**Response:** We thank the reviewer for this positive evaluation. No changes were required in the Conclusion.

**Reviewer #1, Minor Point 1: Data Availability:** While a Google Drive link is provided, PLOS ONE typically requires data to be in a permanent repository, such as Figshare or Dryad, unless there are specific privacy concerns. The authors should confirm if this link is permanent.

**Response:** We thank the reviewer for this helpful observation. We agree that a permanent repository is preferable to a shared Google Drive link. We have therefore deposited the anonymized dataset underlying the findings of this study, together with the data dictionary, in Zenodo. The Data Availability Statement has been updated accordingly.

The updated Data Availability Statement is:

“The anonymized dataset underlying the findings of this study, together with the data dictionary, is publicly available in Zenodo at https://doi.org/10.5281/zenodo.21197178.”

**Reviewer #1, Minor Point 2: Terminology:** Ensure that “non-system-based” is used consistently to describe non-standardized pathways to avoid confusion.

**Response:** We thank the reviewer for this recommendation. To avoid confusion, we revised the Methods section to explicitly define non-standardized access as informal or non-system-based mechanisms. We also reviewed the manuscript to improve consistency in the use of “standardized access,” “non-standardized access,” and “non-system-based mechanisms.”

**Reviewer #1, Minor Point 3: Clarity:** In Table 3, the note defining direct-access specialties includes “Family Medicine,” which is an important detail for understanding the Ecuadorian context.

**Response:** We thank the reviewer for this observation. We revised the Results section and Table 3 to ensure that Family Medicine is consistently included among the direct-access specialties. The direct-access specialties are now consistently described as Dentistry, General Medicine, Family Medicine, and Psychology.

**Response to Reviewer #2**

**Reviewer #2:** Manuscript accepted having minor typing errors. Kindly accept the corrected manuscripts. Manuscripts meet the criteria for PLOS ONE, research ethics and after corrections, the manuscripts may be published.

**Response:** We thank Reviewer #2 for the positive evaluation of our manuscript and for recommending acceptance after minor corrections. We carefully reviewed the manuscript and corrected minor typographical and formatting issues. These included correcting “An classic work” to “A classic work,” harmonizing terminology related to standardized and non-standardized access, correcting the reference to Table 3, improving punctuation in the description of direct-access specialties, and correcting minor capitalization and formatting details in the figure caption and tables.

We again thank the Academic Editor and reviewers for their time and constructive feedback. We believe that the revised version addresses all comments and requirements.

Sincerely,

Marcelo Armijos Briones
On behalf of all authors
