# Supplementary material for: Waiting time for scheduled outpatient specialist consultations by access pathway in public hospitals in Ecuador
Source: PLoS One. 2026 Sep 17;21(9):e0349550. doi: 10.1371/journal.pone.0349550 (PMC13585269; doi:10.1371/journal.pone.0349550)
Supplement: Peer review history

# Peer review: Decision Letter 0

**DOI:** 10.1371/journal.pone.0349550.r001

**Authors:** António Machado

21 Jun 2026

Please include the following items when submitting your revised manuscript:

- A rebuttal letter that responds to each point raised by the academic editor and reviewer(s). You should upload this letter as a separate file labeled 'Response to Reviewers'.
- A marked-up copy of your manuscript that highlights changes made to the original version. You should upload this as a separate file labeled 'Revised Manuscript with Track Changes'.
- An unmarked version of your revised paper without tracked changes. You should upload this as a separate file labeled 'Manuscript'.

If you would like to make changes to your financial disclosure, please include your updated statement in your cover letter. Guidelines for resubmitting your figure files are available below the reviewer comments at the end of this letter.

If applicable, we recommend that you deposit your laboratory protocols in protocols.io to enhance the reproducibility of your results. Protocols.io assigns your protocol its own identifier (DOI) so that it can be cited independently in the future. For instructions see: https://journals.plos.org/plosone/s/submission-guidelines#loc-laboratory-protocols. Additionally, PLOS ONE offers an option for publishing peer-reviewed Lab Protocol articles, which describe protocols hosted on protocols.io. Read more information on sharing protocols at https://plos.org/protocols?utm_medium=editorial-email&utm_source=authorletters&utm_campaign=protocols.

We look forward to receiving your revised manuscript.

Kind regards,

António Machado

Academic Editor

PLOS ONE

Journal requirements:

When submitting your revision, we need you to address these additional requirements.

1.Please ensure that your manuscript meets PLOS ONE's style requirements, including those for file naming. The PLOS ONE style templates can be found at

https://journals.plos.org/plosone/s/file?id=wjVg/PLOSOne_formatting_sample_main_body.pdf and

https://journals.plos.org/plosone/s/file?id=ba62/PLOSOne_formatting_sample_title_authors_affiliations.pdf

2. Please note that funding information should not appear in any section or other areas of your manuscript. We will only publish funding information present in the Funding Statement section of the online submission form. Please remove any funding-related text from the manuscript.

3. Thank you for stating the following in the Competing Interests section:

“The authors have declared that no competing interests exist.”

We note that one or more of the authors are employed by a commercial company: Pvalor SAS, Portoviejo – Ecuador

1.        Please provide an amended Funding Statement declaring this commercial affiliation, as well as a statement regarding the Role of Funders in your study. If the funding organization did not play a role in the study design, data collection and analysis, decision to publish, or preparation of the manuscript and only provided financial support in the form of authors' salaries and/or research materials, please review your statements relating to the author contributions, and ensure you have specifically and accurately indicated the role(s) that these authors had in your study. You can update author roles in the Author Contributions section of the online submission form.

Please also include the following statement within your amended Funding Statement.

“The funder provided support in the form of salaries for authors [insert relevant initials], but did not have any additional role in the study design, data collection and analysis, decision to publish, or preparation of the manuscript. The specific roles of these authors are articulated in the ‘author contributions’ section.”

If your commercial affiliation did play a role in your study, please state and explain this role within your updated Funding Statement.

2. Please also provide an updated Competing Interests Statement declaring this commercial affiliation along with any other relevant declarations relating to employment, consultancy, patents, products in development, or marketed products, etc.

Within your Competing Interests Statement, please confirm that this commercial affiliation does not alter your adherence to all PLOS ONE policies on sharing data and materials by including the following statement: "This does not alter our adherence to  PLOS ONE policies on sharing data and materials.” (as detailed online in our guide for authors http://journals.plos.org/plosone/s/competing-interests) . If this adherence statement is not accurate and  there are restrictions on sharing of data and/or materials, please state these. Please note that we cannot proceed with consideration of your article until this information has been declared.

Please include both an updated Funding Statement and Competing Interests Statement in your cover letter. We will change the online submission form on your behalf.

4. We note you have included a table to which you do not refer in the text of your manuscript. Please ensure that you refer to Table 1 in your text; if accepted, production will need this reference to link the reader to the Table.

Additional Editor comments:

I am pleased to inform you that both reviewers only requested minor revisions for future publication endorsement. Please carefully answer both reviewers' concerns and rectify the manuscript following their comments.

Thank you and best regards,

António Machado

[Note: HTML markup is below. Please do not edit.]

Reviewers' comments:

**Comments to the Author**

1. Is the manuscript technically sound, and do the data support the conclusions?

Reviewer #1: Yes

Reviewer #2: Yes

**********

2. Has the statistical analysis been performed appropriately and rigorously?

Reviewer #1: Yes

Reviewer #2: Yes

**********

3. Have the authors made all data underlying the findings in their manuscript fully available?

The PLOS Data policy

Reviewer #1: Yes

Reviewer #2: Yes

**********

4. Is the manuscript presented in an intelligible fashion and written in standard English?

Reviewer #1: Yes

Reviewer #2: Yes

**********

Reviewer #1: General Overview

The manuscript investigates a critical and often overlooked aspect of health equity: how different access pathways (standardized vs. non-standardized/informal) influence waiting times for specialist care in a public hospital system. Using a large sample (n = 4,436), the authors provide robust empirical evidence that informal pathways systematically result in shorter wait times, particularly for direct-access specialties. This study is highly relevant for policymakers in Latin America and other regions facing similar health system fragmentation.

Specific Comments by Section

1. Title and Abstract (Pages 1–3)

Title (Page 1): The title is descriptive and accurately reflects the study's scope.

Abstract (Pages 2–3, Lines 40–71): The abstract provides a comprehensive summary of the objective, methods, and key findings.

Comment: The abstract clearly highlights the adjusted median difference of -3.2 days and the significant disparity in direct-access specialties (-15.0 days). This clarity is a major strength.

2. Introduction (Pages 3–5, Lines 77–121)

Contextualization (Pages 3–4): The authors effectively frame waiting time as a non-monetary rationing mechanism and establish that delays can lead to disease progression.

Conceptual Framework (Pages 4–5): The introduction of "social connections" as a determinant of timeliness is well-supported by the cited literature regarding Latin American health system fragmentation.

Recommendation: On Page 4 (Line 94), the authors mention that patient-level evidence remains limited in the region. This study successfully bridges that gap.

3. Methods (Pages 6–9, Lines 122–208)

Design and Setting (Page 6): The study uses a cross-sectional hospital survey conducted between July and October 2024.

Definitions (Pages 7–8):

Standardized access is clearly defined as the institutional referral pathway.

Non-standardized access includes informal routes through friends, family, or other personal networks.

Statistical Analysis (Pages 8–9): The use of the Mann–Whitney U test for skewed distributions and quantile regression (τ = 0.50) for adjusted median differences is statistically sound for this type of data.

Ethics (Page 9): The study received appropriate ethics committee approval (CEISH-ITSUP code 1718079732) and obtained informed consent.

4. Results (Pages 10–14, Lines 210–284)

Demographics (Pages 10–11): Table 1 shows that women accounted for 63.5% of the sample and the majority identified as Mestizo.

Key Findings (Page 11, Lines 231–234): The overall median waiting time was 30 days for standardized access versus 25 days for non-standardized access, a statistically significant difference (p < 0.001).

Stratification (Pages 13–14, Lines 268–277): Table 3 presents the most striking finding: in direct-access specialties (e.g., Dentistry, Psychology), the median difference was -15.0 days.

Comment: The presentation of Figure 1 (violin plot) effectively visualizes the right-skewed distribution of waiting times across both groups.

5. Discussion (Pages 15–20, Lines 286–417)

Interpretation (Pages 15–16): The authors correctly identify that non-standardized pathways may reflect the mobilization of "connection" social capital to bypass administrative hurdles.

Equity Implications (Page 17): The discussion highlights a major risk: patients following formal routes (who were referred by a physician) may wait longer than those using informal routes based on self-perceived (and potentially inaccurate) needs.

Limitations (Pages 19–20, Lines 396–408): The manuscript appropriately addresses limitations, including recall bias, the cross-sectional design, and the exclusion of patients who never successfully obtained an appointment.

6. Conclusion (Page 20, Lines 410–417)

Final Summary: The conclusion is supported by the data, stating that timeliness depends partly on factors outside the formal scheduling process.

Recommendation: The call for greater transparency in appointment allocation is a logical and actionable policy recommendation based on these findings.

Reviewer Recommendation

Verdict: Accept with Minor Revisions.

Minor Points for the Authors:

Data Availability (Page 7): While a Google Drive link is provided, PLOS ONE typically requires data to be in a permanent repository (like Figshare or Dryad) unless there are specific privacy concerns. The authors should confirm if this link is permanent.

Terminology (Page 3, Line 51): Ensure that "non-system-based" is used consistently to describe non-standardized pathways to avoid confusion.

Clarity (Page 14, Line 281): In Table 3, the note defining direct-access specialties includes "Family Medicine," which is an important detail for understanding the Ecuadorian context

Reviewer #2: Manuscript accepted having minor typing errors. Kindly accept the corrected manuscripts. Manuscripts meet the criteria for PLOS ONE, research ethics and after corrections, the manuscripts may be published.

**********

what does this mean?). If published, this will include your full peer review and any attached files.

If you choose “no”, your identity will remain anonymous but your review may still be made public.

**Do you want your identity to be public for this peer review?**  For information about this choice, including consent withdrawal, please see our Privacy Policy

Reviewer #1: No

Reviewer #2: No

**********

[NOTE: If reviewer comments were submitted as an attachment file, they will be attached to this email and accessible via the submission site. Please log into your account, locate the manuscript record, and check for the action link "View Attachments". If this link does not appear, there are no attachment files.]

To ensure your figures meet our technical requirements, please review our figure guidelines: https://journals.plos.org/plosone/s/figures

You may also use PLOS’s free figure tool, NAAS, to help you prepare publication quality figures: https://journals.plos.org/plosone/s/figures#loc-tools-for-figure-preparation.

NAAS will assess whether your figures meet our technical requirements by comparing each figure against our figure specifications.

Attachment Submitted filename: Reviewer comment.docx

Attachment Submitted filename: Corrected PONE-D-26-21785.pdf

# Author comment: Author response to Decision Letter 1

**DOI:** 10.1371/journal.pone.0349550.r002

11 Jul 2026

Response to the Academic Editor and Journal Requirements

Editor’s requirement 1: Please ensure that your manuscript meets PLOS ONE’s style requirements, including those for file naming.

Response: We thank the Academic Editor for this reminder. We have reviewed the revised files according to PLOS ONE’s formatting and file-naming requirements. We are submitting the required files separately: a response letter, a revised manuscript with tracked changes, and a clean revised manuscript.

Editor’s requirement 2: Please note that funding information should not appear in any section or other areas of your manuscript. We will only publish funding information present in the Funding Statement section of the online submission form. Please remove any funding-related text from the manuscript.

Response: We thank the Academic Editor for this clarification. We have removed the funding-related text from the manuscript. Specifically, the previous “Funding” section was deleted from the revised manuscript. The Funding Statement will be provided only in the corresponding section of the online submission form and in the cover letter, as requested.

The updated Funding Statement is as follows:

Updated Funding Statement:

The authors received no specific funding for this work. Pvalor SAS provided support in the form of salary for author NAB, but did not have any additional role in the study design, data collection and analysis, decision to publish, or preparation of the manuscript. The specific role of this author is articulated in the author contributions section.

Editor’s requirement 3: Please provide an amended Funding Statement declaring the commercial affiliation, as well as a statement regarding the Role of Funders in your study. Please also provide an updated Competing Interests Statement declaring this commercial affiliation.

Response: We thank the Academic Editor for this request. We have updated the Funding Statement as indicated above. We have also updated the Competing Interests Statement to declare the commercial affiliation of author NAB with Pvalor SAS and to confirm that this affiliation does not alter our adherence to PLOS ONE policies on sharing data and materials.

Updated Competing Interests Statement:

I have read the journal’s policy and the authors of this manuscript have the following competing interests: Nicole Armijos Bazurto is employed by Pvalor SAS, Portoviejo, Ecuador. This commercial affiliation did not influence the study design, data collection and analysis, decision to publish, or preparation of the manuscript. This does not alter our adherence to PLOS ONE policies on sharing data and materials. The remaining authors declare no competing interests.

Editor’s requirement 4: We note you have included a table to which you do not refer in the text of your manuscript. Please ensure that you refer to Table 1 in your text; if accepted, production will need this reference to link the reader to the Table.

Response: We thank the Academic Editor for identifying this issue. We have added an explicit reference to Table 1 in the Results section, immediately before the table. The revised text now reads:

“Participant characteristics and waiting times by access pathway are shown in Table 1.”

Response to Reviewer #1

We thank Reviewer #1 for the careful and positive evaluation of our manuscript and for recognizing the relevance of this study for health equity and health system policy in Latin America.

Reviewer #1, General Overview: The manuscript investigates a critical and often overlooked aspect of health equity: how different access pathways influence waiting times for specialist care in a public hospital system. Using a large sample, the authors provide robust empirical evidence that informal pathways systematically result in shorter wait times, particularly for direct-access specialties. This study is highly relevant for policymakers in Latin America and other regions facing similar health system fragmentation.

Response: We thank the reviewer for this positive assessment. We appreciate the recognition of the relevance of the study and its contribution to the literature on waiting times, access pathways, and health equity in public hospital systems.

Reviewer #1, Title and Abstract: The title is descriptive and accurately reflects the study’s scope. The abstract provides a comprehensive summary of the objective, methods, and key findings. The abstract clearly highlights the adjusted median difference of −3.2 days and the significant disparity in direct-access specialties.

Response: We thank the reviewer for this positive comment. No changes were required in the title or abstract.

Reviewer #1, Introduction: The authors effectively frame waiting time as a non-monetary rationing mechanism and establish that delays can lead to disease progression. The introduction of “social connections” as a determinant of timeliness is well supported by the cited literature. The study successfully bridges the gap in patient-level evidence in the region.

Response: We thank the reviewer for this assessment. No substantive changes were required in the Introduction. However, as part of the minor typographical revision, we corrected the phrase “An classic work” to “A classic work.”

Reviewer #1, Methods: Standardized access is clearly defined as the institutional referral pathway. Non-standardized access includes informal routes through friends, family, or other personal networks. The use of the Mann–Whitney U test and quantile regression is statistically sound. The study received appropriate ethics committee approval and obtained informed consent.

Response: We thank the reviewer for this positive assessment of the methodological and statistical approach. To further improve clarity and address the reviewer’s terminology comment below, we revised the exposure definition to explicitly state that non-standardized access refers to informal or non-system-based mechanisms. The revised text in the Methods section now reads:

“Non-standardized access was defined as obtaining the appointment through informal or non-system-based mechanisms, including through a friend or family member or through a person who was not a friend or family member. Throughout the manuscript, non-standardized access refers to these informal or non-system-based mechanisms.”

Reviewer #1, Results: Table 1 shows the demographic characteristics of the sample. The overall median waiting time was 30 days for standardized access versus 25 days for non-standardized access. Table 3 presents the most striking finding: in direct-access specialties, the median difference was −15.0 days. Figure 1 effectively visualizes the right-skewed distribution of waiting times.

Response: We thank the reviewer for this positive comment. In response to the Academic Editor’s request, we added a direct reference to Table 1 in the Results section. We also revised the description of direct-access specialties in the Results section to include Family Medicine consistently.

Reviewer #1, Discussion: The authors correctly identify that non-standardized pathways may reflect the mobilization of “connection” social capital to bypass administrative hurdles. The discussion highlights a major risk: patients following formal routes may wait longer than those using informal routes based on self-perceived needs. The limitations are appropriately addressed.

Response: We thank the reviewer for this assessment. No substantive changes were required in the Discussion.

Reviewer #1, Conclusion: The conclusion is supported by the data, stating that timeliness depends partly on factors outside the formal scheduling process. The call for greater transparency in appointment allocation is a logical and actionable policy recommendation.

Response: We thank the reviewer for this positive evaluation. No changes were required in the Conclusion.

Reviewer #1, Minor Point 1: Data Availability: While a Google Drive link is provided, PLOS ONE typically requires data to be in a permanent repository, such as Figshare or Dryad, unless there are specific privacy concerns. The authors should confirm if this link is permanent.

Response: We thank the reviewer for this helpful observation. We agree that a permanent repository is preferable to a shared Google Drive link. We have therefore deposited the anonymized dataset underlying the findings of this study, together with the data dictionary, in Zenodo. The Data Availability Statement has been updated accordingly.

The updated Data Availability Statement is:

“The anonymized dataset underlying the findings of this study, together with the data dictionary, is publicly available in Zenodo at https://doi.org/10.5281/zenodo.21197178.”

Reviewer #1, Minor Point 2: Terminology: Ensure that “non-system-based” is used consistently to describe non-standardized pathways to avoid confusion.

Response: We thank the reviewer for this recommendation. To avoid confusion, we revised the Methods section to explicitly define non-standardized access as informal or non-system-based mechanisms. We also reviewed the manuscript to improve consistency in the use of “standardized access,” “non-standardized access,” and “non-system-based mechanisms.”

Reviewer #1, Minor Point 3: Clarity: In Table 3, the note defining direct-access specialties includes “Family Medicine,” which is an important detail for understanding the Ecuadorian context.

Response: We thank the reviewer for this observation. We revised the Results section and Table 3 to ensure that Family Medicine is consistently included among the direct-access specialties. The direct-access specialties are now consistently described as Dentistry, General Medicine, Family Medicine, and Psychology.

Response to Reviewer #2

Reviewer #2: Manuscript accepted having minor typing errors. Kindly accept the corrected manuscripts. Manuscripts meet the criteria for PLOS ONE, research ethics and after corrections, the manuscripts may be published.

Response: We thank Reviewer #2 for the positive evaluation of our manuscript and for recommending acceptance after minor corrections. We carefully reviewed the manuscript and corrected minor typographical and formatting issues. These included correcting “An classic work” to “A classic work,” harmonizing terminology related to standardized and non-standardized access, correcting the reference to Table 3, improving punctuation in the description of direct-access specialties, and correcting minor capitalization and formatting details in the figure caption and tables.

Attachment Submitted filename: Response to Reviewers.docx

# Peer review: Decision Letter 1

**DOI:** 10.1371/journal.pone.0349550.r003

**Authors:** Ehsan Zarei

26 Aug 2026

Dear Dr. Armijos Briones,

Thank you for submitting your manuscript to PLOS One. After careful consideration, we feel that it has merit but does not fully meet PLOS One’s publication criteria as it currently stands. Therefore, we invite you to submit a revised version of the manuscript that addresses the points raised during the review process.

Please submit your revised manuscript by Oct 25 2026 11:59PM. If you will need more time than this to complete your revisions, please reply to this message or contact the journal office at plosone@plos.org. When you're ready to submit your revision, log on to https://www.editorialmanager.com/pone/ and select the 'Submissions Needing Revision' folder to locate your manuscript file.

- A letter that responds to each point raised by the academic editor and reviewer(s). You should upload this letter as a separate file labeled 'Response to Reviewers'.
- A marked-up copy of your manuscript that highlights changes made to the original version. You should upload this as a separate file labeled 'Revised Manuscript with Track Changes'.
- An unmarked version of your revised paper without tracked changes. You should upload this as a separate file labeled 'Manuscript'.

If you would like to make changes to your financial disclosure, please include your updated statement in your cover letter. Guidelines for resubmitting your figure files are available below the reviewer comments at the end of this letter.

If applicable, we recommend that you deposit your laboratory protocols in protocols.io to enhance the reproducibility of your results. Protocols.io assigns your protocol its own identifier (DOI) so that it can be cited independently in the future. For instructions see: https://journals.plos.org/plosone/s/submission-guidelines#loc-laboratory-protocols. Additionally, PLOS One offers an option for publishing peer-reviewed Lab Protocol articles, which describe protocols hosted on protocols.io. Read more information on sharing protocols at https://plos.org/protocols?utm_medium=editorial-email&utm_source=authorletters&utm_campaign=protocols.

As the corresponding author, your ORCID iD is verified in the submission system and will appear in the published article. PLOS supports the use of ORCID, and we encourage all coauthors to register for an ORCID iD and use it as well. Please encourage your coauthors to verify their ORCID iD within the submission system before final acceptance, as unverified ORCID iDs will not appear in the published article. *Only*  the individual author can complete the verification step; PLOS staff *cannot*  verify ORCID iDs on behalf of authors.

We look forward to receiving your revised manuscript.

Kind regards,

Ehsan Zarei

Academic Editor

PLOS One

Journal Requirements:

If the reviewer comments include a recommendation to cite specific previously published works, please review and evaluate these publications to determine whether they are relevant and should be cited. There is no requirement to cite these works unless the editor has indicated otherwise.

Additional Editor Comments:

Dear Authors,

Thank you for your manuscript. I have three methodological concerns that need to be addressed.

1. Hospital clustering

Your data are clustered by hospital. Patients within the same hospital are more similar to each other than to patients in other hospitals due to shared scheduling practices, specialist availability, and administrative procedures. Your current models do not account for this clustering, which violates the independence assumption of your regression models. This can lead to incorrect standard errors and invalid inference.

Recommendation: Please reanalyse all regression models accounting for hospital clustering.

2. Specialty and hospital as covariates

Your primary waiting-time model does not include specialty or hospital as covariates. Both are important potential confounders. Specialty affects waiting times and referral patterns, and hospital affects capacity and scheduling policies. These factors could bias your estimates.

Recommendation: Please adjust your primary model for both specialty and hospital, or provide a strong justification for not doing so.

3. Interaction test

You stratified the analysis by referral requirement and concluded that the effect of access pathway is larger in direct-access specialties. However, comparing estimates from two separate stratified models is not sufficient to conclude that the effect differs between groups.

Recommendation: Please formally test whether the effect of access pathway differs by referral requirement. Fit a single model that allows you to test this interaction, and report the result.

4. Selection Bias — Discussion

You correctly acknowledge in the limitations that your sample only includes patients who successfully attended a scheduled consultation. However, you do not discuss the implications of this bias for your interpretation.

Recommendation: In the Discussion section, please add a paragraph explaining that:

Your estimates represent a lower bound of the true waiting time difference, because patients with the longest waits may have dropped out before attending

Your findings only generalize to patients who successfully navigated the system to attendance, not to those who never obtained an appointment

Future studies should capture unsuccessful attempts to access care

[Note: HTML markup is below. Please do not edit.]

Reviewers' comments:

Reviewer's Responses to Questions

**Comments to the Author**

Reviewer #1: All comments have been addressed

Reviewer #2: (No Response)

**********

2. Is the manuscript technically sound, and do the data support the conclusions?

Reviewer #1: Yes

Reviewer #2: Yes

**********

3. Has the statistical analysis been performed appropriately and rigorously?

Reviewer #1: Yes

Reviewer #2: Yes

**********

4. Have the authors made all data underlying the findings in their manuscript fully available?

The PLOS Data policy

Reviewer #1: Yes

Reviewer #2: Yes

**********

5. Is the manuscript presented in an intelligible fashion and written in standard English?

Reviewer #1: Yes

Reviewer #2: Yes

**********

Reviewer #1: I confirm that the authors have adequately addressed all the questions raised in the previous review. The manuscript has been improved accordingly, and I believe it is now suitable for publication. There are no further concerns or comments from my side.

Reviewer #2: Manuscripts meet the criteria for PLOS ONE. Recommend the manuscript to be published. Kindly process the manuscript to be published

**********

what does this mean?). If published, this will include your full peer review and any attached files.

If you choose “no”, your identity will remain anonymous but your review may still be made public.

**Do you want your identity to be public for this peer review?**  For information about this choice, including consent withdrawal, please see our Privacy Policy

Reviewer #1: No

Reviewer #2: **Yes:** Makou Nnene Melia

**********

[NOTE: If reviewer comments were submitted as an attachment file, they will be attached to this email and accessible via the submission site. Please log into your account, locate the manuscript record, and check for the action link "View Attachments". If this link does not appear, there are no attachment files.]

To ensure your figures meet our technical requirements, please review our figure guidelines: https://journals.plos.org/plosone/s/figures

You may also use PLOS’s free figure tool, NAAS, to help you prepare publication quality figures: https://journals.plos.org/plosone/s/figures#loc-tools-for-figure-preparation.

NAAS will assess whether your figures meet our technical requirements by comparing each figure against our figure specifications.

# Author comment: Author response to Decision Letter 2

**DOI:** 10.1371/journal.pone.0349550.r004

30 Aug 2026

Response to the Academic Editor and Reviewers

Manuscript ID: PONE-D-26-21785R1

Title: Waiting time for scheduled outpatient specialist consultations by access pathway in public hospitals in Ecuador

Dear Dr. Zarei and Reviewers,

We sincerely thank the Academic Editor and both reviewers for their careful evaluation of our revised manuscript. We are grateful for the reviewers’ confirmation that the previous comments were adequately addressed and that the manuscript is suitable for publication. In response to the Academic Editor’s additional methodological concerns, we conducted the requested reanalyses and revised the Abstract, Methods, Results, Tables 2 and 3, Discussion, and Conclusion. Below, we provide a point-by-point response.

In addition, because the analyses were re-run using the dataset corresponding to the final revised analyses, we updated the Zenodo deposit and the Data Availability Statement. The anonymized minimal dataset underlying the findings of this study, together with the data dictionary, is publicly available in Zenodo at https://doi.org/10.5281/zenodo.22166322.

Response to the Academic Editor

Comment 1: Hospital clustering

“Your data are clustered by hospital. Patients within the same hospital are more similar to each other than to patients in other hospitals due to shared scheduling practices, specialist availability, and administrative procedures. Your current models do not account for this clustering, which violates the independence assumption of your regression models. This can lead to incorrect standard errors and invalid inference.

Recommendation: Please reanalyse all regression models accounting for hospital clustering.”

Response: We thank the Academic Editor for identifying this important issue. We reanalysed all regression models using hospital-clustered sandwich variance estimators. This correction was applied to the crude and adjusted logistic regressions, the primary median regression, and the interaction median regression. All clustered models used 26 hospital clusters, a CR1 finite-sample correction, and confidence intervals and hypothesis tests based on a t distribution with 25 degrees of freedom.

The Statistical analysis subsection has been revised to describe the hospital-clustered inference. Table 2 and the corresponding Results paragraph have also been updated with hospital-clustered confidence intervals. The logistic-regression point estimates remained unchanged, but several confidence intervals widened after accounting for within-hospital dependence. In particular, age, Amazonian region, and household income quintiles Q1 and Q2 no longer showed clear adjusted associations with non-standardized access. The associations for rural residence, fourth-level education, and household income quintile Q4 remained statistically supported, while the association for female sex was borderline after clustering.

During this reanalysis, we also identified and corrected a transcription inconsistency in the previously reported crude odds ratio for household income quintile Q1. The crude odds ratio was corrected from 1.61 to 1.36; the adjusted odds ratio remained unchanged at 1.55.

The revised Methods text states:

“Because participants were clustered within hospitals, all regression models used hospital-clustered sandwich variance estimators. We applied a CR1 finite-sample correction and based confidence intervals and hypothesis tests on a t distribution with 25 degrees of freedom, corresponding to 26 hospital clusters. For the median-regression models, the clustered sandwich covariance matrix was based on quantile scores and a residual-density estimate.”

Comment 2: Specialty and hospital as covariates

“Your primary waiting-time model does not include specialty or hospital as covariates. Both are important potential confounders. Specialty affects waiting times and referral patterns, and hospital affects capacity and scheduling policies. These factors could bias your estimates.

Recommendation: Please adjust your primary model for both specialty and hospital, or provide a strong justification for not doing so.”

Response: We agree with the Academic Editor and have adjusted the primary median regression model for both individual specialty and hospital. The revised model included age, sex, education, geographic region, ethnicity, household income quintile, area of residence, individual-specialty fixed effects, and hospital fixed effects. Hospital-clustered inference was applied as described above. All 4,436 participants were retained in the final model.

After this adjustment, non-standardized access was associated with a 3.6-day shorter conditional median waiting time compared with standardized access (adjusted median difference -3.6 days, 95% CI -6.2 to -1.0; p = 0.008). The magnitude was similar to the previously reported estimate of -3.2 days, although the confidence interval was wider after accounting for specialty, hospital, and within-hospital dependence.

The Abstract, Statistical analysis subsection, Results, and opening paragraph of the Discussion have been updated accordingly. The revised Results text states:

“In a median regression model adjusted for age, sex, education, geographic region, ethnicity, household income quintile, area of residence, specialty, and hospital, and using hospital-clustered inference, non-standardized access was associated with a shorter conditional median waiting time than standardized access (adjusted median difference -3.6 days, 95% CI -6.2 to -1.0; p = 0.008).”

Comment 3: Interaction test

“You stratified the analysis by referral requirement and concluded that the effect of access pathway is larger in direct-access specialties. However, comparing estimates from two separate stratified models is not sufficient to conclude that the effect differs between groups.

Recommendation: Please formally test whether the effect of access pathway differs by referral requirement. Fit a single model that allows you to test this interaction, and report the result.”

Response: We thank the Academic Editor for this clarification. We fitted a single median regression model containing an interaction between access pathway and specialty referral-requirement stratum. The model included the same sociodemographic covariates, individual-specialty fixed effects, hospital fixed effects, and hospital-clustered inference as the primary model. Because referral requirement was determined by individual specialty, the referral-requirement main effect was absorbed by the individual-specialty fixed effects, while the interaction term remained identifiable and was formally tested.

In the adjusted interaction model, non-standardized access was associated with a 7.9-day shorter conditional median waiting time in direct-access specialties (adjusted median difference -7.9 days, 95% CI -13.7 to -2.1; p = 0.010) and a 3.1-day shorter conditional median waiting time in referral-required specialties (adjusted median difference -3.1 days, 95% CI -5.9 to -0.2; p = 0.036). However, the interaction term was not statistically significant (interaction estimate -4.8 days, 95% CI -11.2 to 1.6; p = 0.136).

We therefore revised the Abstract, Methods, Results, Table 3, Discussion, and Conclusion to avoid concluding that the association was statistically different between the two referral-requirement strata. The revised manuscript now states that the adjusted point estimate was numerically larger in direct-access specialties, but that the formal interaction test did not provide clear evidence of effect modification by referral requirement.

The revised Results text states:

“However, the interaction between access pathway and referral-requirement stratum was not statistically significant (interaction estimate -4.8 days, 95% CI -11.2 to 1.6; p = 0.136), providing no clear evidence that the association differed between the two strata.”

Comment 4: Selection bias — Discussion

“You correctly acknowledge in the limitations that your sample only includes patients who successfully attended a scheduled consultation. However, you do not discuss the implications of this bias for your interpretation.

Recommendation: In the Discussion section, please add a paragraph explaining that:

Your estimates represent a lower bound of the true waiting time difference, because patients with the longest waits may have dropped out before attending.

Your findings only generalize to patients who successfully navigated the system to attendance, not to those who never obtained an appointment.

Future studies should capture unsuccessful attempts to access care.”

Response: We agree with the Academic Editor and have substantially expanded the discussion of selection bias in the Limitations subsection. The revised text explains that the analytic sample was conditioned on successful attendance and did not include individuals who failed to obtain an appointment, abandoned the process, did not attend the scheduled consultation, or sought care outside the public system.

We now explicitly state that, because patients experiencing the longest waits may have abandoned the process before attendance, the observed waiting-time difference likely represents a conservative lower bound of the true difference among all individuals who initially sought specialist care. We also clarify that the findings apply to adults who successfully attended scheduled specialist consultations in participating public hospitals and should not be generalized to all individuals seeking specialist care.

Finally, the Conclusion now recommends that future studies use longitudinal referral and scheduling records to follow patients from referral initiation through appointment completion and capture unmet demand, unsuccessful attempts to obtain appointments, non-attendance, abandonment, and movement between public and private care.

The revised Limitations text includes the following statement:

“Because patients experiencing the longest waits may have abandoned the process before attendance, our observed waiting-time difference likely represents a conservative lower bound of the true difference among all patients who initially sought specialist care. This interpretation is especially plausible if dropout was more frequent among patients experiencing long waits through standardized pathways. Nevertheless, because unsuccessful attempts were not observed, the precise magnitude of this selection bias cannot be quantified from the available data.”

We also updated the remaining limitations to reflect the new analyses. Specifically, we removed the previous statement that hospital-level heterogeneity had not been modelled and now acknowledge that, although hospital fixed effects and hospital-clustered inference were used, residual confounding by unmeasured differences in capacity, referral implementation, and scheduling policies may remain. We also clarified that all 4,436 participants had complete data for the variables included in the final regression models.

Response to Reviewer #1

Reviewer #1 confirmed that all comments from the previous review round had been adequately addressed and that the manuscript was suitable for publication.

Response: We sincerely thank Reviewer #1 for the careful re-evaluation of our manuscript and for confirming that the previous comments were adequately addressed. We appreciate the reviewer’s positive assessment and recommendation for publication. No additional changes were requested by Reviewer #1.

Response to Reviewer #2

Reviewer #2 confirmed that the manuscript met the criteria for PLOS ONE and recommended publication.

Response: We sincerely thank Reviewer #2 for the positive evaluation and recommendation for publication. No additional changes were requested by Reviewer #2.

We again thank the Academic Editor and reviewers for their constructive comments. We believe that the requested reanalyses and corresponding revisions have strengthened the methodological rigor, transparency, and interpretation of the manuscript.

Sincerely,

Marcelo Armijos Briones

On behalf of all authors

Attachment Submitted filename: Response to the Academic Editor and Reviewers.pdf

# Editor report: Decision Letter 2

**DOI:** 10.1371/journal.pone.0349550.r005

**Authors:** Ehsan Zarei

1 Sep 2026

Waiting time for scheduled outpatient specialist consultations by access pathway in public hospitals in Ecuador

PONE-D-26-21785R2

Dear Dr. Armijos Briones,

We’re pleased to inform you that your manuscript has been judged scientifically suitable for publication and will be formally accepted for publication once it meets all outstanding technical requirements.

Within one week, you’ll receive an e-mail detailing the required amendments. When these have been addressed, you’ll receive a formal acceptance letter and your manuscript will be scheduled for publication.

An invoice will be generated when your article is formally accepted. Please note, if your institution has a publishing partnership with PLOS and your article meets the relevant criteria, all or part of your publication costs will be covered. Please make sure your user information is up-to-date by logging into Editorial Manager at Editorial Manager® and clicking the ‘Update My Information' link at the top of the page. For questions related to billing, please contact billing support.

If your institution or institutions have a press office, please notify them about your upcoming paper to help maximize its impact. If they’ll be preparing press materials, please inform our press team as soon as possible -- no later than 48 hours after receiving the formal acceptance. Your manuscript will remain under strict press embargo until 2 pm Eastern Time on the date of publication. For more information, please contact onepress@plos.org.

Kind regards,

Ehsan Zarei

Academic Editor

PLOS One

Additional Editor Comments (optional):

Reviewers' comments:

# Editor report: Acceptance letter

**DOI:** 10.1371/journal.pone.0349550.r006

**Authors:** Ehsan Zarei

PONE-D-26-21785R2

PLOS One

Dear Dr. Armijos Briones,

I'm pleased to inform you that your manuscript has been deemed suitable for publication in PLOS One. Congratulations! Your manuscript is now being handed over to our production team.

At this stage, our production department will prepare your paper for publication. This includes ensuring the following:

* All references, tables, and figures are properly cited

* All relevant supporting information is included in the manuscript submission,

* There are no issues that prevent the paper from being properly typeset

You will receive further instructions from the production team, including instructions on how to review your proof when it is ready. Please keep in mind that we are working through a large volume of accepted articles, so please give us a few days to review your paper and let you know the next and final steps.

Lastly, if your institution or institutions have a press office, please let them know about your upcoming paper now to help maximize its impact. If they'll be preparing press materials, please inform our press team within the next 48 hours. Your manuscript will remain under strict press embargo until 2 pm Eastern Time on the date of publication. For more information, please contact onepress@plos.org.

You will receive an invoice from PLOS for your publication fee after your manuscript has reached the completed accept phase. If you receive an email requesting payment before acceptance or for any other service, this may be a phishing scheme. Learn how to identify phishing emails and protect your accounts at https://explore.plos.org/phishing.

If we can help with anything else, please email us at customercare@plos.org.

Thank you for submitting your work to PLOS One and supporting open access.

Kind regards,

PLOS One Editorial Office Staff

on behalf of

Dr. Ehsan Zarei

Academic Editor

PLOS One
